# Very-Low-Energy Ketogenic Therapy Modulates the Metabolic–Antioxidant Axis in Patients with Obesity and Type 2 Diabetes: A Non-Randomized Clinical Trial

**DOI:** 10.3390/antiox15070844

**Published:** 2026-07-04

**Authors:** Sabrina Tini, Stefano Celano, Stella Pigni, Elena De Palma, Hilal Irem Ozdemir, Tommaso Raiteri, Alessandro Antonioli, Jessica Baima, Valentina Antoniotti, Marina Caputo, Paolo Marzullo, Flavia Prodam

**Affiliations:** 1 Department of Translational Medicine, University of Piemonte Orientale, 28100 Novara, Italy; sabrina.tini@uniupo.it (S.T.); 20032250@studenti.uniupo.it (E.D.P.); 20062747@studenti.uniupo.it (H.I.O.); tommaso.raiteri@uniupo.it (T.R.); valentina.antoniotti@uniupo.it (V.A.); paolo.marzullo@med.uniupo.it (P.M.); 2 Department of Health Science, University of Piemonte Orientale, 28100 Novara, Italy; stefano.celano@uniupo.it (S.C.); pignistella@gmail.com (S.P.); alessandro.antonioli@uniupo.it (A.A.); jessica.baima@uniupo.it (J.B.); marina.caputo@uniupo.it (M.C.); 3 Unit of Endocrinology, University of Piemonte Orientale, 28100 Novara, Italy

**Keywords:** very-low-energy ketogenic diet, Mediterranean diet, obesity, type 2 diabetes, inflammatory cytokines, antioxidant enzymes

## Abstract

**Background**: Oxidative stress and chronic inflammation contribute to the pathogenesis of obesity and type 2 diabetes (T2D), yet the effects of dietary interventions on endogenous antioxidant defences remain poorly defined. This is a non-randomized study evaluates the effects of very-low-energy ketogenic therapy (VLEKT), compared with a Mediterranean diet (MedD) and a control group, on antioxidants, metabolic, and inflammatory markers. **Materials and Methods**: Thirty adults with obesity and T2D were assigned to VLEKT (*n* = 10), MedD (*n* = 10), or control (*n* = 10) for 90 days. Metabolic parameters, inflammatory cytokines, superoxide dismutase (SOD) and glutathione peroxidase (GPx) activities were assessed. Longitudinal changes were analyzed using linear mixed models. **Results**: VLEKT exhibited significant reductions in body weight, fat mass, HbA1c, and HOMA-IR. SOD activity increased in the VLEKT group, whereas no significant changes were observed in MedD. Changes in SOD were inversely associated with changes in HOMA-IR. GPx showed a less consistent response pattern, while inflammatory markers did not differ between groups. **Conclusions**: VLEKT was associated with substantial metabolic improvement accompanied by a selective modulation of antioxidant enzyme activity. The increase in SOD activity and its association with HOMA-IR suggest a link between metabolic and redox adaptations in subjects with obesity and T2D.

## 1. Introduction

Oxidative stress, defined as an imbalance between reactive oxygen species (ROS) generation and antioxidant defences, has been widely recognized as a common pathophysiological driver of obesity, type 2 diabetes (T2D), and metabolic syndrome. A number of studies have demonstrated that these conditions share a state of chronic low-grade inflammation, accompanied by excessive production of ROS and impaired endogenous antioxidant defence systems, collectively creating a self-perpetuating cycle that underlies metabolic dysfunction and disease progression [1,2,3,4,5]. Indeed, the interplay between oxidative stress and inflammation is well established, with both processes contributing to insulin resistance and the development of metabolic and cardiovascular complications [6,7,8,9].

Redox imbalance can contribute to the development of insulin resistance by disrupting the phosphorylation of insulin-signalling proteins, activating stress-sensitive and pro-inflammatory signalling pathways, and inducing mitochondrial damage [10,11]. Furthermore, emerging evidence indicates that oxidative stress actively modulates adipose tissue remodelling through redox-sensitive mechanisms, influencing both adipocyte differentiation and the capacity for healthy adipose expansion [12,13].

In patients with obesity and T2D, reduced activity of key antioxidant enzymes such as superoxide dismutase (SOD), catalase (CAT), and glutathione peroxidase (GPx) has been consistently reported, alongside elevated levels of pro-inflammatory cytokines and oxidative stress biomarkers, including interleukin-1β (IL-1β) and -6 (IL-6), tumour necrosis factor-alpha (TNF-α), lipid peroxidation markers such as malondialdehyde (MDA), and protein carbonyls [14,15,16]. Moreover, an upregulation of phagocytic NADPH oxidase has been documented in subjects with T2D and metabolic syndrome, and correlates with increased oxidative stress [17,18,19]. Therefore, antioxidant and inflammatory markers represent potentially relevant biomarkers for assessing cardiometabolic risk and response to treatments [3,4].

Dietary patterns are among the most powerful modulators of oxidative stress and inflammation. Western (WD) and fast-food diets, characterized by a high intake of refined sugars, saturated fats, and ultra-processed foods, are positively associated with pro-inflammatory and oxidative stress biomarkers, whereas Mediterranean and plant-based diets show inverse associations [20]. The WD is mechanistically linked to increased oxidative stress through multiple interconnected pathways. Nutritional stress induced by a high-fat, high-carbohydrate intake, together with increased exposure to dietary advanced glycation end-products (AGEs) and certain food additives, promotes oxidative burden through increased mitochondrial ROS generation and NADPH-oxidase activation, impaired antioxidant capacity (e.g., reduced SOD activity), and activation of several pro-inflammatory pathways (e.g., NF-κB, RAGE, NLRP3 inflammasome, and Toll-like receptors signalling pathways) [3,21,22,23,24,25]. In addition, Western dietary patterns promote gut microbiota dysbiosis that serves as a critical mediator between diet, inflammation, and oxidative stress [21,26].

By contrast, the Mediterranean diet (MedD) is regarded as a classical antioxidant dietary pattern [27]. Rich in fibre, polyphenols, polyunsaturated fatty acids, and other bioactive compounds, the Med diet has been shown to improve lipid and glycemic profiles, mitigate systemic inflammation, reduce oxidative damage to both lipids and DNA, and enhance endogenous antioxidant defences in individuals with T2D and metabolic syndrome [28,29,30]. Notably, such protective effects are also partly mediated through improved mitochondrial efficiency [31].

More recently, the ketogenic diet (KD), in particular as a very-low-energy protocol (VLEKT), has been increasingly used as a therapeutic strategy for obesity and obesity-related conditions, including T2D and metabolic dysfunction-associated liver disease (MASLD) [32]. Beyond its established effects on weight loss and glycemic control, KD may also improve inflammatory status and reduce oxidative stress in individuals with obesity [33]. Ketone bodies, particularly β-hydroxybutyrate, act not only as alternative energy substrates but also as signalling molecules capable of exerting anti-inflammatory effects, enhancing mitochondrial function, and promoting resistance to oxidative stress [33,34,35,36]. Of note, KDs appear to exert a paradoxical effect on redox homeostasis, initially inducing mild oxidative stress that triggers adaptive (hormetic) responses, ultimately enhancing antioxidant defences and reducing chronic oxidative damage [36]. However, clinical evidence regarding the impact of KD on antioxidant systems remains scarce. Moreover, few studies have directly compared the Mediterranean-style diet and VLEKT in terms of their effects on oxidative stress, antioxidant markers and inflammatory cytokines [37]. Furthermore, limited data are available from longitudinal studies integrating circulating antioxidant enzymes and biochemical markers of inflammation with clinical and metabolic outcomes in well-characterized cohorts, including appropriate control groups.

Therefore, the aim of this study is to investigate the effects of a VLEKT compared with an energy-restricted MedD on key antioxidant enzymes (SOD, GPx) and pro-inflammatory cytokines, and to evaluate their relationship with clinical, metabolic, and body composition parameters in patients with obesity and T2D. Additionally, we compared these findings with those observed in a control cohort of patients not undergoing any structured dietary intervention apart from general dietary advice, to better characterize diet-related changes in redox and inflammatory state.

## 2. Materials and Methods

### 2.1. Study Design

**Figure 1:**
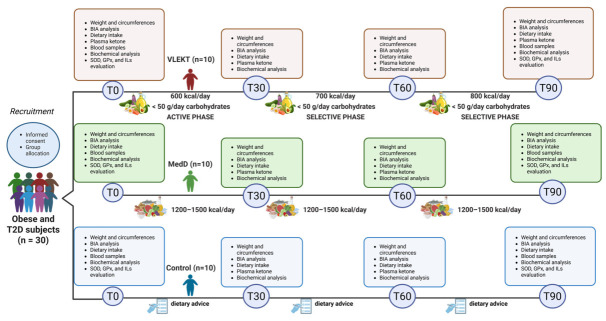
Study design and timeline of the dietary intervention protocol. Participants with obesity and T2D were allocated to one of three groups: VLEKT, hypocaloric Mediterranean diet (MedD), or control. Weight, waist and hip circumferences, BIA analysis, biochemical, dietary assessments, and antioxidant evaluation were performed at baseline and at the end of the study (T0–T90). Weight and body circumferences, BIA analysis, biochemical and dietary assessments, were performed every 30 days until the end of the intervention period (T90). The VLEKT protocol included an active phase (T0–T30) followed by a selective phase with progressive reintroduction of natural protein meals (T30–T90).

This is a 3-month prospective, longitudinal pilot study. Participants were recruited at the Endocrinology Unit (Maggiore della Carità Hospital, Novara) and assigned to three study groups. Two groups received a structured dietary intervention, one very-low-energy ketogenic therapy (VLEKT) and the other an energy-restricted Mediterranean diet (MedD). The third group served as a control and received general dietary advice without a structured dietary intervention. Clinical, anthropometric, biochemical, and dietary assessments were performed at baseline (T0) and at 30-day intervals until study end (T90). Intermediate visits were primarily used for clinical monitoring, nutritional counselling, dietary adherence assessment, and safety evaluation, whereas the primary analytical comparison was defined from baseline to study end (T0-T90). This approach was adopted to compare the effects of the active and selective phase of VLEKT with other dietary regimens and to allow minimum time for dietary efficacy, particularly for the MedD group. A schematic representation of the study design is reported in Figure 1.

### 2.2. Ethical Considerations and Privacy

The study was conducted in accordance with the World Medical Association Declaration of Helsinki 2013, and data were collected and managed in compliance with the EU General Data Protection Regulation (GDPR 2016/679). The study protocol was registered on ClinicalTrials.gov (NCT05975541 and NCT05275608) and approved by the local Ethics Committee (Protocol No. CE 277/2022, approval: 21 March 2023; and Protocol No. 196/2021, approval: 2 February 2022). All participants provided written informed consent prior to enrolment. This research used plasma and serum samples from 30 participants stored in the UPO Biobank (https://biobank.uniupo.it/, accessed on 2 July 2026).

### 2.3. Study Population and Dietary Interventions

A total of 30 subjects (15 males and 15 females), aged 25–65 years, with obesity (BMI 30–40 kg/m^2^) and previously diagnosed T2D (mean disease duration: 5.6 ± 4.3 years) were recruited. Obesity and T2D were confirmed from clinical records according to WHO and ADA criteria, respectively [38,39]. T2D diagnosis was based on fasting plasma glucose ≥126 mg/dL and/or HbA1c ≥6.5%, or 2 h plasma glucose ≥200 mg/dL during OGTT. At baseline, all participants were receiving a stable glucose-lowering therapy, mainly consisting of metformin and/or GLP-1 receptor agonists, with no treatment variations throughout the study. No patients were receiving insulin analogues. In the VLEKT group, SGLT2 inhibitors were discontinued 7 days before the baseline assessment (T0) in participants receiving this treatment (1/10), according to current clinical guidelines [40]. Lipid-lowering therapy was also used by all participants (Table S1). Exclusion criteria included secondary forms of obesity, endocrine disorders, pregnancy, severe or complicated medical conditions, ongoing treatment with systemic corticosteroids or immunosuppressive agents, and use of antibiotics, probiotics, or prebiotics within the 15 days preceding study enrolment.

Participants were assigned to the VLEKT, the energy-restricted MedD, or control groups, according to clinical evaluation, patient preference, and feasibility of adherence to the assigned nutritional protocol, for three months.

Subjects assigned to the VLEKT group followed a structured 3-month nutritional programme providing approximately 30–40 g/day of carbohydrates, 25–30 g/day of fat, 1.2–1.5 g/kg of protein based on ideal body weight, and 22–30 g/day of fibre, with a total energy intake ranging from 600 to 800 kcal/day. The VLEKT protocol consisted of two phases. During the active phase, corresponding to the first 30 days, participants consumed exclusively meal-replacement products, four per day for women and five per day for men, together with low-glycemic-index vegetables at main meals. During the selective phase, starting from day 31, natural protein meals, including eggs, white meat, and fish, were progressively reintroduced while meal replacements were gradually reduced. After completion of the 12-week study period, a progressive carbohydrate reintroduction phase was planned to support dietary transition and prevent weight regain. Vitamin and mineral supplementation were provided throughout the intervention according to established guidelines [41].

Participants in the MedD group followed an energy-restricted Mediterranean diet protocol providing 45–55% of total energy from carbohydrates, 25–30% of lipids and 1–1.2 g/kg from proteins. The dietary plan was designed to provide a caloric deficit of 500 kcal/day relative to basal energy requirements, which was estimated using an algorithm provided by Tanita MC-780 MA (Tanita Europe B.V., Amsterdam, The Netherlands).

Participants in the control group were followed for 3 months without receiving any structured dietary intervention or specific dietary targets, although general nutritional advice on healthy diets were provided during follow-up visits for ethical reasons.

### 2.4. Clinical Assessment and Dietary Adherence Monitoring

Anthropometric, clinical, and biochemical assessments were performed at baseline (T0) and at 30-day intervals until study end (T90), alongside nutritional counselling and dietary monitoring. At each visit, patients were asked to report any adverse events occurring during the intervention. In the VLEKT group, adherence was assessed using a five-point study-specific questionnaire developed by the research team to evaluate satiety, consumption of foods outside the prescribed protocol, and self-reported symptoms and perceptions related to the ketogenic programme. In addition, capillary β-hydroxybutyrate concentrations were measured every three days during the first 30 days using a portable device (GlucoMen Aero K, Menarini Diagnostics, Florence, Italy), whereas urinary ketones were assessed by routine urine analysis during follow-up visits throughout the 90-day intervention. These measurements were used to quantitatively document the achievement of nutritional ketosis during the active phase of the VLEKT protocol, while urinary ketone testing provided qualitative monitoring of ketone production during the subsequent selective phase. In the MedD group, dietary adherence was assessed using 24 h dietary recalls and the PREDIMED questionnaire [42,43]. In the control group, 24 h dietary recalls and the PREDIMED questionnaire were used to monitor changes in dietary habits over time and to describe variations in energy intake relative to baseline. Due to the different dietary structure and prescribed targets across the three study groups, dietary-energy changes were calculated using group-specific reference values. In the VLEKT group, adherence to the nutritional intervention was assessed by monitoring ketone levels. In the MedD group, a ±10% deviation from the prescribed caloric intake was used to determine adequate adherence to the assigned intervention. In the control group, a ±10% deviation from baseline reported energy intake was used to evaluate changes from baseline. To avoid the risk of excessive fatigue, hypoglycemia, dehydration, and reduced adherence to the VLEKT protocol, participants were instructed not to initiate structured physical exercise during the intervention period. To ensure comparability between groups and minimize exercise-related bias, the same recommendation was applied to all groups.

### 2.5. Anthropometric and Body Composition Evaluation

Clinical and anthropometric evaluations were performed at the predefined timepoints. Assessments included a brief medical history, measurement of systolic and diastolic blood pressure, and anthropometric measurements, including height, body weight, waist circumference, and hip circumference. Body mass index (BMI) was calculated as body weight divided by height squared (kg/m^2^), and waist-to-height ratio (WHtR) as waist circumference divided by height. Body composition, including fat mass, fat-free mass, skeletal muscle mass, phase angle, basal metabolic rate (BMR), and body fluid distribution, was assessed by bioelectrical impedance analysis (BIA; Tanita MC-780, Tanita Europe B.V., Amsterdam, The Netherlands).

### 2.6. Blood Sampling and Biochemical Assessment

Fasting blood samples were collected at baseline and every 30 days until the end of the dietary intervention to assess glucose and lipid profile [fasting plasma glucose (FPG), fasting plasma insulin (FPI), glycated hemoglobin (HbA1c%), total-cholesterol (T-c), high-density cholesterol (HDL-c) and triglycerides (TG)]. LDL-cholesterol (LDL-c) was calculated with the Friedewald formula [44]. Insulin resistance was estimated using HOMA-IR, calculated accordingly [45]. Additional blood samples were collected, at baseline and at the end of the study to obtain serum and plasma fractions, and stored for future analyses according to standardized procedures.

### 2.7. Inflammatory Markers

To assess systemic inflammation, Neutrophils-to-lymphocytes ratio (NLR) was calculated as follows:

Neutrophil-to-lymphocyte ratio (NLR) = neutrophil count (10^3^/μL)/lymphocyte count (10^3^/μL).

Pro-inflammatory cytokines (CCL2, IL-1β, IL-18, CXCL5, IL-6 and TNF-α) were evaluated on serum samples, collected and stored under standardized conditions at −80 °C until analysis. Cytokines were quantified using Luminex^®^ Human Premixed Multi-Analyte Assay (catalogue number: LXSAHM, Abingdon, OX143NB, UK) and Luminex 200™ analyzer (Bio-Techne, Minneapolis, MN, USA), according to the manufacturer’s instructions. Briefly, serum samples were incubated with magnetic microparticles pre-coated with analyte-specific antibodies, followed by biotinylated detection antibodies and streptavidin-phycoerythrin. Fluorescence signals were then acquired using the Luminex 200™ system, and analyte concentrations were calculated from standard curves generated for each cytokine. Values reported as out of range (<OOR) were considered non-quantifiable and were not included in the statistical analyses. Since all IL-1β values were reported as <OOR, IL-1β was excluded from inferential analyses.

### 2.8. Antioxidant Activity

SOD and GPx activity were evaluated in plasma samples collected in EDTA tubes and stored at −80 °C under standardized conditions until analysis. Briefly, SOD activity was assessed using a colorimetric inhibition assay (Superoxide Dismutase Activity Assay Kit, ab65354, Abcam, Cambridge, UK), based on the ability of SOD to scavenge superoxide radicals generated by xanthine oxidase, thereby inhibiting the reduction in water-soluble tetrazolium salt (WST) to formazan. Plasma samples were incubated with the reaction mixture for 20 min at 37 °C, and absorbance was measured at 440 nm. SOD activity was calculated as inhibition rate (%) according to the manufacturer’s instruction.

GPx activity was measured using a colorimetric coupled enzyme assay (Glutathione Peroxidase Activity Assay Kit, ab102535, Abcam, Cambridge, UK). The assay is based on the GPx-mediated reduction in cumene hydroperoxide, coupled with the oxidation of glutathione and its subsequent recycling by glutathione reductase, with concomitant consumption of NADPH. Briefly, plasma samples were incubated with a reaction mixture containing glutathione, glutathione reductase, and NADPH, followed by addition of cumene hydroperoxide to initiate the GPx reaction. The decrease in absorbance at 340 nm, reflecting NADPH oxidation, was measured using a microplate reader. GPx activity was calculated according to the manufacturer’s instructions and expressed as mU/mL.

### 2.9. Statistical Analysis

Statistical analyses were conducted using baseline and post-intervention data (T0–T90) to compare changes among the three study groups.

Continuous variables were assessed for normality using the Shapiro–Wilk test. Data are presented as mean ± standard deviation (SD) for normally distributed variables and median (interquartile range, IQR) for non-normally distributed variables. Baseline differences among groups were assessed using one-way ANOVA or Kruskal–Wallis tests, as appropriate.

Longitudinal changes in antioxidants, metabolic, inflammatory, and body composition outcomes were evaluated using separate linear mixed-effects models for each outcome. Each model included time, group, and their interaction (time × group) as fixed effects, with participant included as a random intercept. Age and sex were included as covariates in all models, together with the baseline value of the specific outcome under investigation. The time × group interaction was used to assess between-group differences in changes over time. Model assumptions were verified through residual diagnostics; variables for which residuals showed departure from normality were log-transformed prior to analysis.

As exploratory analysis, associations between changes in antioxidant markers and metabolic, inflammatory, or dietary variables were explored using Spearman correlation analyses and multivariable linear regression models with robust standard errors, as appropriate. Within-group changes in PREDIMED scores from baseline to post-intervention were assessed using the Wilcoxon signed-rank test. To identify multivariate response patterns linking antioxidant, metabolic and inflammatory changes, principal component analysis (PCA) was performed on delta (Δ) values of selected variables.

Statistical analyses were performed using Stata (version 19.5; StataCorp, College Station, TX, USA). PCA analyses and related biplot visualization were conducted in R (version 4.5.3; R Foundation for Statistical Computing, Vienna, Austria). Additional figures were generated using GraphPad Prism (version 10.4.0; GraphPad Software, San Diego, CA, USA). Statistical significance was set at α = 0.05.

## 3. Results

A total of 30 participants (50% females and 50% males) aged 55.1 ± 6.8 years with obesity (BMI 35.4 ± 3.8 kg/m^2^) and T2D (HbA1c 6.7 ± 0.9%) were recruited. Participants were equally distributed across the three groups, with 10 subjects in the VLEKT group, 10 in the MedD group, and 10 in the control group. Baseline, clinical, metabolic characteristics, circulating inflammatory markers and antioxidant enzyme levels were largely comparable across groups. However, significant baseline differences were observed for age, BMI, fat mass, HbA1c and GPx levels. Compared with the control and MedD groups, the VLEKT group was younger and showed higher BMI, fat mass, and GPx levels. HbA1c also differed significantly at baseline, with the control group showing the highest values. These imbalances were accounted for in subsequent adjusted models, which included age, sex and baseline values of the dependent variable. Baseline characteristics are represented in Table 1.

### 3.1. Effects of Dietary Interventions on Anthropometric and Metabolic Parameters

To characterize the metabolic effects of the interventions, adjusted LMMs were used to assess changes in anthropometric and biochemical parameters from T0 to T90 (Table 2 and Table S2). VLEKT showed marked reductions in body weight, BMI, fat mass and WHtR (*p* interaction = 0.001). Of note, FFM also decreased in the VLEKT group, while no significant changes in body composition were observed in the MedD or control groups.

Regarding glycemic control and insulin resistance, VLEKT showed significant reductions in FPG (*p* interaction = 0.007), HbA1c (*p* time × group interaction = 0.014) and HOMA-IR (*p* time × group interaction < 0.001). No significant changes in these parameters were observed in the MedD or control groups. Among lipid parameters, no significant between-group differences were observed in the lipid profile, with the exception of a borderline trend for HDL-C (*p* time × group interaction = 0.077). Within the VLEKT group, total cholesterol and triglycerides showed reductions from baseline that did not reach between-group significance (Table S3).

### 3.2. Dietary Adherence

Given the similar anthropometric and biochemical trajectories observed between the MedD and control groups, dietary adherence was further analyzed to better characterize the nutritional exposure during follow-up. In the VLEKT group, protocol compliance was adequate, as indicated by a mean blood β-hydroxybutyrate concentration of 1.83 ± 0.42 mmol/L, with individual mean values ranging from 1.20 to 2.51 mmol/L, consistent with sustained nutritional ketosis throughout the study. Conversely, the MedD and control groups showed a different adherence pattern. PREDIMED scores increased significantly in both the MedD and control groups (*p* = 0.020 and *p* = 0.023, respectively), with no significant difference in the magnitude of change between groups, indicating a comparable improvement in Mediterranean diet adherence over time although the control group received only structured advice (Table S4). As the MedD intervention was designed as a caloric-restricted dietary protocol, deviations between reported energy intake at T90 and the individualized prescribed energy target were calculated to assess adherence to the assigned nutritional protocol. At T90, two out of 10 MedD participants reported an energy intake within ±10% of the prescribed caloric target. In the control group, deviations were calculated relative to baseline energy intake to assess whether participants maintained or modified their dietary habits during follow-up. Overall, four out of 10 control participants remained within ±10% of their baseline energy intake (Tables S5 and S6).

### 3.3. Inflammatory Response to Dietary Interventions

At T90, no significant differences for any of the cytokines examined were noted among groups. Within-group analyses showed a trend toward relative reductions in IL-6 in both the control (−24.4%; *p* = 0.060) and the VLEKT groups (−21.3%; *p* = 0.098). A similar trend was observed for IL-18 in the VLEKT group (*p* = 0.064). In addition, NLR, a surrogate inflammatory index, did not change significantly between groups, while platelet count decreased significantly in both the VLEKT group (β = −27.9) and the control group (β = −21.4), but not in the MedD group (*p* = 0.021).

### 3.4. Changes in Circulating Antioxidant Enzymes After 90 Days of Dietary Interventions

**Figure 2:**
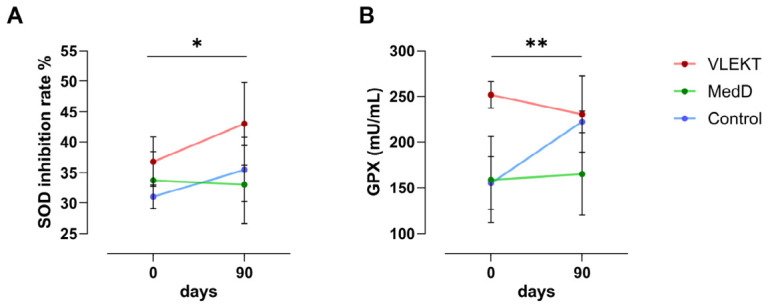
Changes in circulating antioxidant enzymes from T0 to T90 across study groups. (**A**) SOD inhibition rate at baseline (T0) and study end (T90) in the VLEKT, MedD and control groups. (**B**) GPx levels at baseline (T0) and study end (T90) in the VLEKT, MedD and control groups. Dots represent individual participants, bars indicate group means, and error bars denote 95% confidence intervals. Between-group changes are indicated by asterisks (* *p* < 0.05; ** *p* < 0.01). *p* interaction values refer to the group × time interaction derived from adjusted linear mixed-effects models. Abbreviations: VLEKT, very-low-energy ketogenic therapy; MedD, Mediterranean diet; GPx, glutathione peroxidase.

At T90, LMMs revealed divergent patterns of change in antioxidant enzyme levels across groups (Figure 2). SOD inhibition rate increased significantly in both the VLEKT group (β = 6.18, 95% CI [2.44–9.93]) and the control group (β = 4.46, 95% CI [0.72–8.21]), whereas no significant differences were observed in the MedD group (*p* time × group interaction = 0.029).

A contrasting pattern was observed for GPx, which increased significantly from baseline only in the control group (β = 66.78, 95% CI [32.04–101.52]; *p* time × group interaction = 0.002), with no significant within-group changes detected in the VLEKT or MedD groups (Table S3).

### 3.5. PCA-Based Profiling of Metabolic and Inflammatory Changes Across Dietary Groups

**Figure 3:**
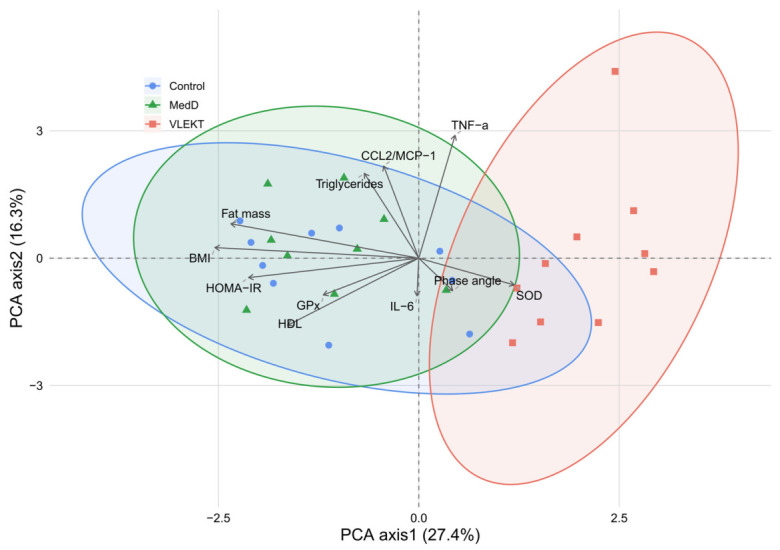
PCA biplot of metabolic, inflammatory, antioxidant and body composition changes. PCA biplot of changes (Δ) in selected metabolic, inflammatory, antioxidant and body composition variables. PC1 and PC2 explained 27.4% and 16.3% of total variance, respectively. Loadings along PC1 were mainly driven by SOD and phase angle in the opposite direction to fat mass, BMI and HOMA-IR, whereas PC2 was primarily associated with inflammatory markers, particularly TNF-α and MCP-1. Abbreviations: PCA, principal component analysis; PC, principal component; Δ, change from baseline to study end; SOD, superoxide dismutase; BMI, body mass index; HOMA-IR, homeostasis model assessment of insulin resistance; TNF-α, tumour necrosis factor-alpha; MCP-1, monocyte chemoattractant protein-1; GPx, glutathione peroxidase; HDL, high-density lipoprotein cholesterol; IL-6, interleukin-6.

To explore the multivariate metabolic and inflammatory profiles of the three groups, a principal component analysis (PCA) was performed (Figure 3). PCA axis 1 (PC1) and PCA axis 2 explained 27.4% and 16.3% of the total variance, respectively. PC1 was interpreted as a metabolic axis, driven by variables related to insulin resistance and lipid metabolism, while PC2 represented an inflammatory axis, reflecting the contribution of pro-inflammatory cytokines. The biplot revealed a clear separation of the VLEKT group along PC1, whereas the MedD and the control group substantially overlapped. The VLEKT group was distinctly displaced toward positive PC1 values, suggesting a divergent metabolic profile compared to the other two groups, with higher scores along variables such as SOD and phase angle. The control and the MedD groups showed substantial overlap along both axes, clustering predominantly in the negative PC1 region and indicating similar metabolic and inflammatory profiles. Notably, MedD subjects displayed a wider dispersion along PC2, suggesting greater inter-individual variability in inflammatory markers within this group. The observed group separation was statistically confirmed by PERMANOVA (F = 2.99, R^2^ = 0.18, *p* = 0.001).

### 3.6. Associations Between Antioxidant Enzymes with Metabolic and Inflammatory Markers

Although PCA suggested that both SOD and phase angle clustered toward a more favourable metabolic profile, correlation analysis did not show a significant association between changes in SOD and phase angle. In contrast, a moderate inverse correlation was observed between changes in GPx and phase angle (ρ = −0.37, *p* = 0.045). However, this association was not confirmed in adjusted linear regression models, indicating that neither relationship remained significant after accounting for potential confounders. Spearman correlation analyses were then performed to assess whether changes in antioxidant markers were associated with changes in metabolic and inflammatory parameters. Changes in SOD showed a trend toward an inverse association with changes in HOMA-IR (ρ = −0.36, *p* = 0.058), while no significant correlations were observed with changes in fat mass, body weight or triglycerides (Table S7). By contrast, changes in GPx were positively correlated with changes in HOMA-IR (ρ = 0.42, *p* = 0.024) and body weight (ρ = 0.37, *p* = 0.049) (Table S7). Additionally, to further assess these associations, multivariable regression models were fitted separately for changes in SOD and GPx (Tables S8 and S9). Given the substantial overlap between the control and MedD groups in the PCA, the intervention group was modelled as VLEKT versus control and MedD combined. In the model for SOD, changes in HOMA-IR were independently associated with changes in SOD after adjustment for changes in fat mass, intervention group, age, sex and baseline SOD (β = −0.47, *p* = 0.036). In the corresponding model for GPx, no significant associations were observed for changes in fat mass, changes in HOMA-IR or intervention group. Furthermore, to explore whether the observed variations in antioxidant enzyme activity were related to differences in nutritional exposure, changes in caloric intake were correlated with changes in SOD, GPx and inflammatory markers. Changes in caloric intake were not significantly associated with changes in SOD activity (Spearman’s ρ = −0.060, *p* = 0.750), whereas a positive correlation was observed with changes in GPx activity (Spearman’s ρ = 0.444, *p* = 0.015) (Table S10). No significant correlations were observed between antioxidant enzyme activity and inflammatory markers, including circulating cytokines and NLR. Similarly, caloric intake was not significantly associated with inflammatory parameters.

## 4. Discussion

Obesity and T2D are characterized by profound metabolic dysfunction, chronic low-grade inflammation and impaired redox homeostasis [46,47]. Although lifestyle interventions remain the cornerstone of treatment, the extent to which different dietary strategies modulate endogenous antioxidant defences in this clinical setting is still poorly defined. Limited evidence is available on whether VLEKT interventions could be accompanied by specific antioxidant adaptations beyond their established effects on weight loss and glycemic control. In this proof-of-concept study, we showed that VLEKT was associated with the most pronounced rapid metabolic improvement coupled with an increase in SOD activity that seems to be associated with the improvement in insulin resistance markers.

The anthropometric and glucometabolic effects observed after VLEKT are consistent with the established efficacy of these approaches in obesity management. European Association for the Study of Obesity (EASO) describes VLEKT as a structured nutritional strategy characterized by severe energy and carbohydrate restriction, with documented effects on body weight, body composition and glycemic parameters in adults with obesity [32]. In our cohort, VLEKT produced substantial reductions in body weight and fat mass, as expected, paralleled by improvements in FPG, HbA1c and HOMA-IR. These findings support the view that, in patients with obesity and T2D, a highly structured VLEKT intervention exhibited a rapid metabolic improvement. By contrast, the MedD arm showed a blunted anthropometric and glucometabolic effect within 3 months. This finding is not surprising in the short term and should also be interpreted in the context of suboptimal adherence to the prescribed dietary plan which may have reduced its metabolic effects, as suggested by the relatively low dietary adherence in this group. On the other hand, the effects of MedD could be demonstrated in a more prolonged period, in agreement with guidelines that suggest the MedD as one of the best dietary strategies in the management of T2D and obesity [48,49,50]. Given the poor adherence we observed, weight and glycemic trajectories were similar between the MedD and control group.

Pro-inflammatory cytokines did not show significant intervention-related changes. This finding should not necessarily be interpreted as absence of immunometabolic effects. Rather, it may indicate that systemic cytokine concentrations are less sensitive than anthropometric, glycemic or redox markers over the short term, particularly in a small cohort, and in the context of obesity associated with T2D. Rohm et al. emphasize that the inflammatory milieu in obesity and T2D involves complex, multi-cellular interactions within adipose and other metabolically active tissues, whose resolution may not be readily captured by changes in circulating cytokines alone, especially over a 90-day window [47]. Ketone bodies, particularly β-hydroxybutyrate, have been shown to exert anti-inflammatory actions in experimental models, including inhibition of NLRP3 inflammasome activation. However, translating these mechanisms into measurable reductions in circulating cytokines in human dietary studies is not straightforward. Baseline inflammatory status, duration of intervention, degree of weight loss, medication background and assay variability may all influence the detectability of cytokine changes [34,51]. In our cohort, IL-6 showed only non-significant trends toward reduction in the control and VLEKT groups. Similarly, Quetglas-Llabrés et al. observed that meaningful reductions in inflammatory markers in patients following a MedD lifestyle intervention required sustained adherence over 24 months, suggesting that longer follow-up may be necessary to capture diet-induced immunomodulatory effects [52].

A key observation of this study is the divergent behaviour of the two antioxidant enzymes across groups. SOD increased in both the VLEKT and control groups, whereas GPx increased only in the control group, while neither enzyme showed significant changes in the MedD group. However, the increase in SOD should not be interpreted in isolation or considered biologically equivalent across groups, as it occurred within distinct metabolic-redox contexts. In the VLEKT group, SOD increased in parallel with marked improvements in body weight, fat mass, glycemic control and insulin resistance, without a concomitant increase in GPx activity. By contrast, in the control group, SOD increased together with GPx, in the absence of comparable anthropometric and biochemical improvements. Similarly, the increase in GPx observed only in the control group should be interpreted within this broader response pattern, rather than as an isolated marker of improved antioxidant status. Collectively, these data do not indicate a VLEKT-specific enhancement of antioxidant defences, but rather a context-specific modulation of antioxidant enzyme activity, whereby comparable SOD changes may reflect different underlying metabolic adaptations.

This pattern suggests that SOD and GPx should not be interpreted as interchangeable markers of a single “antioxidant status”, but rather as components of a coordinated and context-dependent redox network [46,53]. These differences suggest that the redox response observed after VLEKT may reflect a selective metabolic-redox adaptation, whereas the concomitant increase in SOD and GPx in the control group should be interpreted more cautiously. Although adherence analyses suggest that control participants modified some dietary habits during follow-up, increased SOD and GPx activity cannot be unequivocally interpreted as a favourable redox adaptation. These changes may also reflect compensatory or context-dependent antioxidant responses, behavioural changes during follow-up, and inter-individual variability [16]. Finally, the absence of significant antioxidant enzyme changes in the MedD group may reflect the lack of a sufficiently strong or consistent dietary stimulus, in line with the suboptimal adherence observed in this arm. This distinction is particularly relevant in obesity and T2D, conditions in which oxidative stress contributes to insulin resistance, β-cell dysfunction and dyslipidemia.

Within this context, the association between SOD and HOMA-IR, while remaining exploratory, is one of the most clinically relevant findings of this study. In correlation analyses, changes in SOD showed a trend toward an inverse association with changes in HOMA-IR. This relationship became significant in multivariable regression after adjustment for fat mass change, intervention group, age, sex and baseline value. Thus, the association between SOD and insulin resistance seemed to be not explained solely by adiposity reduction or by allocation to the VLEKT group. From a clinical perspective, this suggests that improvement in insulin resistance may occur in parallel with modulation of antioxidant defence, supporting a close link between redox adaptation and metabolic improvement. The pathophysiological basis for this link is well established, since superoxide anions generated by NADPH oxidase and mitochondrial ROS impair insulin receptor signalling cascades, whereas restoration of SOD activity may partially protect these pathways [46,54]. Furthermore, data from high-fat-diet animal models have demonstrated that improvements in redox homeostasis biomarkers, including SOD activity, correlate with improvements in metabolic syndrome, providing translational support for the associations observed in our cohort [54]. The absence of an independent association between changes in fat mass and changes in SOD further refines this interpretation. VLEKT produced the greatest reduction in fat mass, but the association between SOD and HOMA-IR persisted after accounting for fat mass change. This finding suggests that SOD variation may be more closely related to changes in insulin resistance dynamics than to fat loss per se. This is consistent with the biological model in which oxidative stress and insulin resistance reinforce each other through mitochondrial dysfunction, impaired insulin signalling and chronic metabolic stress [11,55]. In this framework, the association between SOD and HOMA-IR suggests that SOD may track improvement in metabolic homeostasis rather than simply reflecting the magnitude of weight loss.

On the other hand, GPx showed a less consistent relationship with metabolic improvement. Although changes in GPx correlated with changes in HOMA-IR and body weight in Spearman correlation analyses, these associations were not retained in the corresponding multivariable model. Nevertheless, the positive correlation between changes in GPx and body weight indicates that greater weight loss was associated with lower GPx. This observation is consistent with recent dietary weight-loss evidence showing that improvements in metabolic and redox status are not necessarily accompanied by increased GPx activity. In particular, Szlachta et al. reported a significant reduction in erythrocyte GPx after weight reduction, despite concomitant decreases in glucose, HOMA-IR, malondialdehyde and lipofuscin, together with increased glutathione reductase activity, suggesting adaptive remodelling of the glutathione-dependent antioxidant system rather than impaired antioxidant defence [56]. This may reflect a more complex regulation of GPx in obesity and T2D. Experimental and translational studies have shown that GPx, as an extracellular member of the peroxidase family, is dysregulated in obesity and may influence adipose tissue insulin signalling. Moreover, GPx activity can be influenced by baseline antioxidant capacity, inflammatory signals, and inter-individual variability [57,58]. Similarly, Bosch-Sierra et al. observed an improvement in redox status after VLCD-induced weight loss, characterized by reduced mitochondrial ROS and increased glutathione levels, but did not find an increase in GPX1 mRNA expression, further supporting the idea that GPx-related responses may be uncoupled from global metabolic improvement [59]. Finally, Bellanti et al. [60] reported a reduction in serum oxidative damage markers, whereas erythrocyte redox markers, including GPx activity, did not significantly change, suggesting that diet-induced redox improvement may be more evident at the systemic level than within the erythrocyte enzymatic compartment. Therefore, GPx may not behave as a linear marker of short-term metabolic improvement in this setting. In line with this hypothesis, the moderate association between changes in caloric intake and GPx observed in our cohort further suggests that GPx variations may be partly influenced by dietary exposure, energy balance and weight-loss dynamics.

Importantly, the PCA provided a useful integrated view of these findings. The VLEKT group was separated from both the control and MedD groups along a component mainly characterized by metabolic and redox-related variables, whereas the control and MedD groups showed substantial overlap. This multivariate pattern supports the idea that VLEKT may reflect a broader metabolic-redox shift rather than isolated changes in single outcomes. The systematic review by Aleksandrova et al. similarly reported that dietary interventions with high-antioxidant-quality dietary patterns produce multivariate shifts in both oxidative stress and inflammatory biomarker profiles, and that these shifts are most consistently observed when the intervention is sufficiently intensive and sustained [20]. In our study, the relative separation of the VLEKT group along the metabolic-redox axis of the PCA may reflect precisely this type of broader systemic adaptation. Nevertheless, the targeted correlation and regression analyses were essential to qualify the PCA findings. Although SOD and phase angle loaded toward a favourable metabolic-redox direction, their direct association was not significant. GPx showed an inverse correlation with phase angle, but this association was not retained after adjustment.

Our study has several limitations that should be acknowledged. First, the sample size was small, which limits statistical power, especially for cytokine and multivariable analyses. Second, the study was non-randomized, and participants were allocated according to clinical evaluation, patient preference, and expected adherence to the assigned dietary intervention. Although statistical models were adjusted for age, sex, and baseline values of the dependent variables, residual confounding and selection bias cannot be excluded. Therefore, the observed associations should be interpreted as exploratory results rather than as evidence of causal effects. Third, the MeD diet was prescribed as energy-restricted, but suboptimal adherence to the dietary plan likely reduced the effective implementation of the prescribed energy deficit and limited the ability to evaluate the full potential of this intervention. Accordingly, the lack of significant changes in the MedD group should not be interpreted as evidence against the Mediterranean dietary pattern itself. Third, participants in the control group did not receive a structured dietary prescription, but repeated nutritional assessments for ethical reasons and counselling may have influenced dietary behaviour during follow-up, limiting its interpretation as a completely unchanged comparator. Fourth, another important limitation is that the VLEKT intervention differed from the other interventions in several components, including the magnitude of energy restriction, meal-replacement use, vitamin and mineral supplementation, and carbohydrate restriction. Accordingly, the observed metabolic and antioxidant changes cannot be attributed specifically to ketosis or β-hydroxybutyrate per se, but should rather be interpreted as related to the overall structured VLEKT program. Fifth, circulating antioxidant enzymes and cytokines provide only a partial view of redox and inflammatory biology and may not fully capture tissue-level processes. Moreover, direct oxidative damage markers, such as malondialdehyde (MDA), protein carbonyls, and 8-hydroxy-2′-deoxyguanosine (8-OHdG), were not measured. This limits the interpretation of changes in SOD and GPx activities, as we cannot determine whether these antioxidant enzyme responses were accompanied by reductions in lipid, protein, or DNA oxidative damage. Fifth, although baseline medication use did not significantly differ across groups and remained unchanged throughout the study, pharmacological treatments were not included as covariates in the mixed-effects models. Therefore, residual confounding related to background medication cannot be completely excluded. Finally, the relatively short follow-up may have been sufficient to detect metabolic and redox changes but not longer-term inflammatory remodelling. Future studies with larger sample sizes, longer follow-up periods, stricter dietary adherence monitoring, and comprehensive biomarker panels, including oxidative damage products, tissue-level redox markers and specific GPx isoforms, are warranted to confirm and extend these findings.

## 5. Conclusions

In conclusion, our exploratory results highlight that VLEKT was associated with marked improvements in body weight, fat mass and glycemic control in patients with obesity and T2D, accompanied by a distinct modulation of antioxidant markers. Our findings support the hypothesis that dietary interventions may differentially affect metabolic, redox and inflammatory pathways, and suggest that SOD may represent a candidate marker of metabolic-redox adaptation during nutritional therapy. They also underscore the need to move beyond global measures of “antioxidant status” and to consider the specific biological roles and regulatory contexts of individual antioxidant enzymes when interpreting the effects of nutritional interventions in metabolically compromised patients.

## Figures and Tables

**Table 1:** Baseline characteristics of the study population.

Variable	Total (*n* = 30)	Control (*n* = 10)	VLEKT (*n* = 10)	MedD (*n* = 10)	*p*-Value
Clinical and anthropometric					
Age (years)	55.1 ± 6.8	57.7 ± 3.6	50.2 ± 8.2	57.3 ± 5.6	0.017
Female, *n* (%)	15 (50)	5 (50)	5 (50)	5 (50)	1.000
BMI (kg/m^2^)	35.4 ± 3.8	33.0 ± 2.1	39.4 ± 2.9	34.0 ± 2.7	<0.001
Fat mass (kg)	34.5 ± 9.1	30.3 ± 7.2	42.4 ± 8.5	30.7 ± 6.4	0.001
Fat-free mass (kg)	64.8 ± 13.0	60.3 ± 11.9	67.8 ± 15.6	66.3 ± 11.0	0.409
Phase angle (°)	6.22 ± 0.85	6.51 ± 0.85	5.89 ± 0.64	6.26 ± 0.99	0.266
Biochemical					
FPG (mg/dL)	117.5 [104–135]	134.0 [107–142]	118.5 [104–121]	107.0 [90–128]	0.080
FPI(µIU/mL)	18.2 [12.9–26.3]	16.5 [14.6–25.4]	30.0 [18.5–40.3]	14.2 [12.9–24.2]	0.105
HbA1c (%)	6.7 ± 0.9	7.2 ± 1.1	6.6 ± 0.7	6.2 ± 0.6	0.040
HOMA-IR	5.67 [3.24–8.90]	5.47 [3.74–8.84]	7.30 [4.94–11.81]	3.74 [2.86–7.66]	0.362
Total cholesterol (mg/dL)	148.5 ± 37.9	140.5 ± 35.8	151.7 ± 33.0	153.4 ± 46.4	0.725
Triglycerides (mg/dL)	120.5 [90–208]	161.5 [102–208]	116.0 [90–206]	106.0 [85–215]	0.765
HDL-C (mg/dL)	43.1 ± 9.6	40.6 ± 8.1	44.0 ± 12.8	44.7 ± 7.6	0.612
LDL-C (mg/dL)	76.5 ± 29.9	68.6 ± 29.0	79.1 ± 26.2	81.7 ± 35.2	0.598
Inflammatory markers					
IL-6 (pg/mL)	2.79 [2.13–3.46]	2.79 [2.13–3.46]	2.79 [2.13–3.46]	2.79 [2.13–3.46]	0.972
TNF-α (pg/mL)	5.15 ± 1.21	5.43 ± 1.15	5.40 ± 1.42	4.63 ± 0.94	0.248
MCP-1 (pg/mL)	359.5 [333.7–433.0]	372.3 [318.1–542.6]	374.7 [353.0–473.4]	340.7 [266.3–384.3]	0.137
IL-18 (pg/mL)	428.3 [340.7–616.0]	432.6 [384.7–522.9]	492.5 [323.0–616.0]	428.3 [402.2–666.3]	0.971
CXCL5/ENA-78 (pg/mL)	1232.7 [799.8–1828.4]	1402.9 [976.2–3046.8]	1271.1 [799.8–1890.2]	1188.7 [790.9–1546.2]	0.635
NLR	1.91 [1.47–2.40]	1.73 [1.35–4.10]	1.83 [1.45–2.18]	2.13 [1.78–2.44]	0.575
Platelets (×10^9^/L)	261.8 ± 72.1	281.9 ± 73.4	273.7 ± 77.9	229.7 ± 59.7	0.226
Antioxidant markers					
SOD (inh. rate)	32.7 [29.6–36.6]	30.2 [28.8–32.7]	36.6 [33.7–40.3]	32.1 [28.8–35.9]	0.064
GPx (mU/mL)	191.8 [128.7–249.3]	133.9 [128.7–191.5]	249.9 [243.5–263.1]	147.6 [104.6–227.3]	<0.001

Data are presented as mean ± SD or median [IQR], as appropriate. *p*-values derived from one-way ANOVA or Kruskal–Wallis tests; χ^2^/Fisher’s exact test for categorical variables. Abbreviations: BMI, body mass index; FPG, fasting plasma glucose; FPI, fasting plasma insulin; HbA1c, glycated hemoglobin; HOMA-IR, homeostasis model assessment of insulin resistance; HDL-C, high-density lipoprotein cholesterol; LDL-C, low-density lipoprotein cholesterol; NLR, neutrophil-to-lymphocyte ratio; IL-6, interleukin-6; TNF-α, tumour necrosis factor-alpha; MCP-1, monocyte chemoattractant protein-1; IL-18, interleukin-18; CXCL5/ENA-78, C-X-C motif chemokine ligand 5; SOD, superoxide dismutase; GPx, glutathione peroxidase.

**Table 2:** Longitudinal effects of dietary interventions on anthropometric and biochemical parameters.

Variable	Control β (95% CI)	VLEKT β (95% CI)	MedD β (95% CI)	*p* Interaction
Anthropometric				
Weight (kg)	−1.05 (−3.41, 1.31)	−16.22 (−18.58, −13.86)	−1.45 (−3.81, 0.91)	<0.001
BMI (kg/m^2^)	−0.30 (−1.16, 0.56)	−5.90 (−6.76, −5.04)	−0.48 (−1.34, 0.38)	<0.001
WHtR	0.00 (−0.02, 0.03)	−0.08 (−0.10, −0.06)	−0.01 (−0.03, 0.02)	<0.001
Fat mass (kg)	−1.59 (−4.12, 0.94)	−11.57 (−14.10, −9.04)	−0.65 (−3.26, 1.96)	<0.001
Fat-free mass (kg)	0.54 (−0.83, 1.91)	−4.22 (−5.59, −2.85)	−0.54 (−1.95, 0.87)	<0.001
Phase angle (°)	−0.16 (−0.66, 0.34)	−0.16 (−0.38, 0.06)	−0.20 (−0.42, 0.02)	0.966
Metabolic				
ln(FPG)	−0.05 (−0.16, 0.06)	−0.22 (−0.33, −0.11)	0.03 (−0.08, 0.14)	0.007
HbA1c (%)	−0.30 (−0.77, 0.17)	−0.98 (−1.45, −0.51)	−0.02 (−0.49, 0.45)	0.014
HOMA-IR	0.78 (−1.50, 3.06)	−6.12 (−8.40, −3.84)	0.35 (−1.93, 2.63)	<0.001
Total cholesterol (mg/dL)	−13.50 (−29.08, 2.08)	−18.70 (−34.28, −3.12)	−4.20 (−19.78, 11.38)	0.426
HDL-C (mg/dL)	0.50 (−3.10, 4.10)	−4.60 (−8.20, −1.00)	0.50 (−3.10, 4.10)	0.077
LDL-C (mg/dL)	−8.94 (−23.11, 5.23)	−7.24 (−21.41, 6.93)	−4.92 (−19.09, 9.25)	0.925
ln(TG)	−0.20 (−0.41, 0.00)	−0.25 (−0.46, −0.05)	−0.01 (−0.22, 0.19)	0.237

Data are presented as regression coefficients (β) with 95% confidence intervals derived from adjusted linear mixed-effects models. β coefficients represent the estimated change from T0 to T90 within each group. The *p* interaction refers to the group × time interaction term. Models were adjusted for age, sex and baseline values of the dependent variable. Variables showing relevant departures from residual normality were log-transformed before analysis; β coefficients for ln-transformed outcomes are reported on the log scale. Abbreviations: VLEKT, very-low-energy ketogenic therapy; MedD, Mediterranean diet; BMI, body mass index; WHtR, waist-to-height ratio; ln, natural logarithm; FPG, fasting plasma glucose; HbA1c, glycated hemoglobin; HOMA-IR, homeostasis model assessment of insulin resistance; HDL-C, high-density lipoprotein cholesterol; LDL-C, low-density lipoprotein cholesterol; TG, triglycerides.

## Data Availability

The data presented in this study are available on request from the corresponding author due to ethical reasons.

## Supplementary Materials

The following supporting information can be downloaded at: https://www.mdpi.com/article/10.3390/antiox15070844/s1, Table S1. Medication use before enrolment, at baseline and during follow-up according to intervention group; Table S2. Adjusted pairwise contrasts of changes from T0 to T90 for key outcomes across groups; Table S3. Model-adjusted within-group changes from baseline in anthropometric, metabolic, antioxidant, and inflammatory parameters; Table S4. Changes in PREDIMED score in the MedD and control groups; Table S5. Energy adherence to the prescribed Mediterranean dietary intervention at T90; Table S6. Energy intake variation relative to baseline in the control group at T90; Table S7. Spearman correlations of ΔSOD and ΔGPx with metabolic parameters; Table S8. Multivariable linear regression analysis assessing factors associated with changes in SOD; Table S9. Multivariable linear regression analysis assessing factors associated with changes in GPx; Table S10. Association between changes in energy intake and antioxidant enzyme activity.

## Abbreviations

The following abbreviations are used in this manuscript:

VLEKT	Very-low-energy ketogenic therapy
FFM	Fat-free mass
MedD	Low-energy mediterranean diet
BMI	Body mass index
FM	Fat mass
KD	Ketogenic diet
AOU	Azienda Ospedaliero Universitaria
BIA	Bioelectrical impedance analysis
T2D	Type 2 diabetes
SD	Standard deviation
IQR	Interquartile range
GPx	Glutathione peroxidase
SOD	Superoxide dismutase
EASO	European association for the study of obesity
PCA	Principal component analysis
PC1	Principal component axis 1
PC2	Principal component axis 2
HOMA-IR	Homeostasis model assessment of insulin resistance
TNF- α	Tumour necrosis factor alpha
MCP-1	Monocyte chemoattractant protein–1
HDL	High-density lipoprotein cholesterol
IL	Interleukin
FPG	Fasting plasma glucose
FPI	Fasting plasma insulin
HbA1c	Glycated hemoglobin
LDL	Low-density lipoprotein cholesterol
TG	Triglycerides
NLR	Neutrophil-to-lymphocyte ratio
CXCL5/ENA-78	C-X-C motif chemokine ligand 5

## References

[1] Furukawa S., Fujita T., Shimabukuro M., Iwaki M., Yamada Y., Nakajima Y., Nakayama O., Makishima M., Matsuda M., Shimomura I. (2004). Increased Oxidative Stress in Obesity and Its Impact on Metabolic Syndrome. J. Clin. Investig..

[2] Henriksen E.J., Diamond-Stanic M.K., Marchionne E.M. (2011). Oxidative Stress and the Etiology of Insulin Resistance and Type 2 Diabetes. Free Radic. Biol. Med..

[3] Rani V., Deep G., Singh R.K., Palle K., Yadav U.C.S. (2016). Oxidative Stress and Metabolic Disorders: Pathogenesis and Therapeutic Strategies. Life Sci..

[4] Raut S.K., Khullar M. (2023). Oxidative Stress in Metabolic Diseases: Current Scenario and Therapeutic Relevance. Mol. Cell Biochem..

[5] Kawai T., Autieri M.V., Scalia R. (2021). Adipose Tissue Inflammation and Metabolic Dysfunction in Obesity. Am. J. Physiol.-Cell Physiol..

[6] Hauck A.K., Huang Y., Hertzel A.V., Bernlohr D.A. (2019). Adipose Oxidative Stress and Protein Carbonylation. J. Biol. Chem..

[7] Akoumianakis I., Antoniades C. (2019). Impaired Vascular Redox Signaling in the Vascular Complications of Obesity and Diabetes Mellitus. Antioxid. Redox Signal..

[8] Niemann B., Rohrbach S., Miller M.R., Newby D.E., Fuster V., Kovacic J.C. (2017). Oxidative Stress and Cardiovascular Risk: Obesity, Diabetes, Smoking, and Pollution. J. Am. Coll. Cardiol..

[9] Yuan T., Yang T., Chen H., Fu D., Hu Y., Wang J., Yuan Q., Yu H., Xu W., Xie X. (2019). New Insights into Oxidative Stress and Inflammation during Diabetes Mellitus-Accelerated Atherosclerosis. Redox Biol..

[10] Ayer A., Fazakerley D.J., James D.E., Stocker R. (2022). The Role of Mitochondrial Reactive Oxygen Species in Insulin Resistance. Free Radic. Biol. Med..

[11] Feng Z., Tan Z., Lu D. (2025). Mitochondrial Bioenergetics Dysfunction in T2DM: Linking Oxidative Stress to Insulin Resistance. Front. Endocrinol..

[12] Wager J.L., Baker L.G., Scheidl T.B., Yonan S.Z., Colarusso P., Young D., Dufour A., Thompson J.A. (2025). Interleukin-6 from the Adipose Secretome Potentiates Differentiation of Adipose Progenitors through the Activation of Redox Signaling. Am. J. Physiol.-Cell Physiol..

[13] Okuno Y., Fukuhara A., Hashimoto E., Kobayashi H., Kobayashi S., Otsuki M., Shimomura I. (2018). Oxidative Stress Inhibits Healthy Adipose Expansion Through Suppression of SREBF1-Mediated Lipogenic Pathway. Diabetes.

[14] Bigagli E., Lodovici M. (2019). Circulating Oxidative Stress Biomarkers in Clinical Studies on Type 2 Diabetes and Its Complications. Oxidative Med. Cell. Longev..

[15] Vona R., Gambardella L., Cittadini C., Straface E., Pietraforte D. (2019). Biomarkers of Oxidative Stress in Metabolic Syndrome and Associated Diseases. Oxidative Med. Cell. Longev..

[16] Cecerska-Heryć E., Engwert W., Michałów J., Marciniak J., Birger R., Serwin N., Heryć R., Polikowska A., Goszka M., Wojciuk B. (2025). Oxidative Stress Markers and Inflammation in Type 1 and 2 Diabetes Are Affected by BMI, Treatment Type, and Complications. Sci. Rep..

[17] Jansen F., Yang X., Franklin B.S., Hoelscher M., Schmitz T., Bedorf J., Nickenig G., Werner N. (2013). High Glucose Condition Increases NADPH Oxidase Activity in Endothelial Microparticles That Promote Vascular Inflammation. Cardiovasc. Res..

[18] Fortuño A., San José G., Moreno M.U., Beloqui O., Díez J., Zalba G. (2006). Phagocytic NADPH Oxidase Overactivity Underlies Oxidative Stress in Metabolic Syndrome. Diabetes.

[19] DeVallance E., Li Y., Jurczak M.J., Cifuentes-Pagano E., Pagano P.J. (2019). The Role of NADPH Oxidases in the Etiology of Obesity and Metabolic Syndrome: Contribution of Individual Isoforms and Cell Biology. Antioxid. Redox Signal..

[20] Aleksandrova K., Koelman L., Rodrigues C.E. (2021). Dietary Patterns and Biomarkers of Oxidative Stress and Inflammation: A Systematic Review of Observational and Intervention Studies. Redox Biol..

[21] Clemente-Suárez V.J., Beltrán-Velasco A.I., Redondo-Flórez L., Martín-Rodríguez A., Tornero-Aguilera J.F. (2023). Global Impacts of Western Diet and Its Effects on Metabolism and Health: A Narrative Review. Nutrients.

[22] Biobaku F., Ghanim H., Batra M., Dandona P. (2019). Macronutrient-Mediated Inflammation and Oxidative Stress: Relevance to Insulin Resistance, Obesity, and Atherogenesis. J. Clin. Endocrinol. Metab..

[23] Lim S., Won H., Kim Y., Jang M., Jyothi K.R., Kim Y., Dandona P., Ha J., Soo Kim S. (2011). Antioxidant Enzymes Induced by Repeated Intake of Excess Energy in the Form of High-Fat, High-Carbohydrate Meals Are Not Sufficient to Block Oxidative Stress in Healthy Lean Individuals. Br. J. Nutr..

[24] Cepas V., Collino M., Mayo J.C., Sainz R.M. (2020). Redox Signaling and Advanced Glycation Endproducts (AGEs) in Diet-Related Diseases. Antioxidants.

[25] Martínez Leo E.E., Peñafiel A.M., Hernández Escalante V.M., Cabrera Araujo Z.M. (2021). Ultra-Processed Diet, Systemic Oxidative Stress, and Breach of Immunologic Tolerance. Nutrition.

[26] Tini S., Baima J., Pigni S., Antoniotti V., Caputo M., De Palma E., Cerbone L., Grosso F., La Vecchia M., Bona E. (2025). The Microbiota-Diet-Immunity Axis in Cancer Care: From Prevention to Treatment Modulation and Survivorship. Nutrients.

[27] Martini D. (2025). Corrigendum to Mediterranean Diet: Why a New PYRAMID? An Updated Representation of the Traditional Mediterranean Diet by the Italian Society of Human Nutrition (SINU). Nutr. Metab. Cardiovasc. Dis..

[28] Mitjavila M.T., Fandos M., Salas-Salvadó J., Covas M.-I., Borrego S., Estruch R., Lamuela-Raventós R., Corella D., Martínez-Gonzalez M.Á., Sánchez J.M. (2013). The Mediterranean Diet Improves the Systemic Lipid and DNA Oxidative Damage in Metabolic Syndrome Individuals. A Randomized, Controlled, Trial. Clin. Nutr..

[29] Quetglas-Llabrés M.M., Monserrat-Mesquida M., Bouzas C., Gómez C., Mateos D., Ripoll-Vera T., Tur J.A., Sureda A. (2022). Inflammatory and Oxidative Stress Markers Related to Adherence to the Mediterranean Diet in Patients with Metabolic Syndrome. Antioxidants.

[30] Barrea L., Verde L., Colao A., Mandarino L.J., Muscogiuri G. (2025). Medical Nutrition Therapy for the Management of Type 2 Diabetes Mellitus. Nat. Rev. Endocrinol..

[31] Pollicino F., Veronese N., Dominguez L.J., Barbagallo M. (2023). Mediterranean Diet and Mitochondria: New Findings. Exp. Gerontol..

[32] Muscogiuri G., El Ghoch M., Colao A., Hassapidou M., Yumuk V., Busetto L., Obesity Management Task Force (OMTF) of the European Association for the Study of Obesity (EASO) (2021). European Guidelines for Obesity Management in Adults with a Very Low-Calorie Ketogenic Diet: A Systematic Review and Meta-Analysis. Obes. Facts.

[33] Barrea L., Caprio M., Watanabe M., Cammarata G., Feraco A., Muscogiuri G., Verde L., Colao A., Savastano S. (2023). Could Very Low-Calorie Ketogenic Diets Turn off Low Grade Inflammation in Obesity? Emerging Evidence. Crit. Rev. Food Sci. Nutr..

[34] Neudorf H., Little J.P. (2024). Impact of Fasting & Ketogenic Interventions on the NLRP3 Inflammasome: A Narrative Review. Biomed. J..

[35] Rojas-Morales P., Pedraza-Chaverri J., Tapia E. (2020). Ketone Bodies, Stress Response, and Redox Homeostasis. Redox Biol..

[36] Kolb H., Kempf K., Röhling M., Lenzen-Schulte M., Schloot N.C., Martin S. (2021). Ketone Bodies: From Enemy to Friend and Guardian Angel. BMC Med..

[37] Lorenzo P.M., Sajoux I., Izquierdo A.G., Gomez-Arbelaez D., Zulet M.A., Abete I., Castro A.I., Baltar J., Portillo M.P., Tinahones F.J. (2022). Immunomodulatory Effect of a Very-Low-Calorie Ketogenic Diet Compared with Bariatric Surgery and a Low-Calorie Diet in Patients with Excessive Body Weight. Clin. Nutr..

[38] Body Mass Index (BMI). https://www.who.int/data/gho/data/themes/topics/topic-details/GHO/body-mass-index.

[39] Bajaj M., McCoy R.G., Balapattabi K., Bannuru R.R., Bellini N.J., Bennett A.K., Beverly E.A., Briggs Early K., ChallaSivaKanaka S., American Diabetes Association Professional Practice Committee for Diabetes (2026). 2. Diagnosis and Classification of Diabetes: Standards of Care in Diabetes—2026. Diabetes Care.

[40] American Diabetes Association Professional Practice Committee (2025). 5. Facilitating Positive Health Behaviors and Well-Being to Improve Health Outcomes: Standards of Care in Diabetes—2025. Diabetes Care.

[41] Barrea L., Caprio M., Grassi D., Cicero A.F.G., Bagnato C., Paolini B., Muscogiuri G. (2024). A New Nomenclature for the Very Low-Calorie Ketogenic Diet (VLCKD): Very Low-Energy Ketogenic Therapy (VLEKT). Ketodiets and Nutraceuticals Expert Panels: “KetoNut”, Italian Society of Nutraceuticals (SINut) and the Italian Association of Dietetics and Clinical Nutrition (ADI). Curr. Nutr. Rep..

[42] Martínez-González M.A., García-Arellano A., Toledo E., Salas-Salvadó J., Buil-Cosiales P., Corella D., Covas M.I., Schröder H., Arós F., Gómez-Gracia E. (2012). A 14-Item Mediterranean Diet Assessment Tool and Obesity Indexes among High-Risk Subjects: The PREDIMED Trial. PLoS ONE.

[43] Shim J.-S., Oh K., Kim H.C. (2014). Dietary Assessment Methods in Epidemiologic Studies. Epidemiol. Health.

[44] Friedewald W.T., Levy R.I., Fredrickson D.S. (1972). Estimation of the Concentration of Low-Density Lipoprotein Cholesterol in Plasma, without Use of the Preparative Ultracentrifuge. Clin. Chem..

[45] Matthews D.R., Hosker J.P., Rudenski A.S., Naylor B.A., Treacher D.F., Turner R.C. (1985). Homeostasis Model Assessment: Insulin Resistance and β-Cell Function from Fasting Plasma Glucose and Insulin Concentrations in Man. Diabetologia.

[46] Korac B., Kalezic A., Pekovic-Vaughan V., Korac A., Jankovic A. (2021). Redox Changes in Obesity, Metabolic Syndrome, and Diabetes. Redox Biol..

[47] Rohm T.V., Meier D.T., Olefsky J.M., Donath M.Y. (2022). Inflammation in Obesity, Diabetes, and Related Disorders. Immunity.

[48] Evert A.B., Dennison M., Gardner C.D., Garvey W.T., Lau K.H.K., MacLeod J., Mitri J., Pereira R.F., Rawlings K., Robinson S. (2019). Nutrition Therapy for Adults With Diabetes or Prediabetes: A Consensus Report. Diabetes Care.

[49] Aas A.-M., Axelsen M., Churuangsuk C., Hermansen K., Kendall C.W.C., Kahleova H., Khan T., Lean M.E.J., Mann J.I., The Diabetes and Nutrition Study Group (DNSG) of the European Association for the Study of Diabetes (EASD) (2023). Evidence-Based European Recommendations for the Dietary Management of Diabetes. Diabetologia.

[50] Hassapidou M., Vlassopoulos A., Kalliostra M., Govers E., Mulrooney H., Ells L., Salas X.R., Muscogiuri G., Darleska T.H., Busetto L. (2023). European Association for the Study of Obesity Position Statement on Medical Nutrition Therapy for the Management of Overweight and Obesity in Adults Developed in Collaboration with the European Federation of the Associations of Dietitians. Obes. Facts.

[51] Youm Y.-H., Nguyen K.Y., Grant R.W., Goldberg E.L., Bodogai M., Kim D., D’Agostino D., Planavsky N., Lupfer C., Kanneganti T.D. (2015). The Ketone Metabolite β-Hydroxybutyrate Blocks NLRP3 Inflammasome-Mediated Inflammatory Disease. Nat. Med..

[52] Quetglas-Llabrés M.M., Monserrat-Mesquida M., Bouzas C., García S., Argelich E., Casares M., Ugarriza L., Llompart I., Tur J.A., Sureda A. (2024). Impact of Adherence to the Mediterranean Diet on Antioxidant Status and Metabolic Parameters in NAFLD Patients: A 24-Month Lifestyle Intervention Study. Antioxidants.

[53] Jomova K., Alomar S.Y., Alwasel S.H., Nepovimova E., Kuca K., Valko M. (2024). Several Lines of Antioxidant Defense against Oxidative Stress: Antioxidant Enzymes, Nanomaterials with Multiple Enzyme-Mimicking Activities, and Low-Molecular-Weight Antioxidants. Arch. Toxicol..

[54] Di Majo D., Sardo P., Giglia G., Di Liberto V., Zummo F.P., Zizzo M.G., Caldara G.F., Rappa F., Intili G., Van Dijk R.M. (2022). Correlation of Metabolic Syndrome with Redox Homeostasis Biomarkers: Evidence from High-Fat Diet Model in Wistar Rats. Antioxidants.

[55] Veluthakal R., Esparza D., Hoolachan J.M., Balakrishnan R., Ahn M., Oh E., Jayasena C.S., Thurmond D.C. (2024). Mitochondrial Dysfunction, Oxidative Stress, and Inter-Organ Miscommunications in T2D Progression. Int. J. Mol. Sci..

[56] Szlachta B., Birková A., Čižmárová B., Głogowska-Gruszka A., Zalejska-Fiolka P., Dydoń M., Zalejska-Fiolka J. (2024). Erythrocyte Oxidative Status in People with Obesity: Relation to Tissue Losses, Glucose Levels, and Weight Reduction. Antioxidants.

[57] Lee Y.S., Kim A.Y., Choi J.W., Kim M., Yasue S., Son H.J., Masuzaki H., Park K.S., Kim J.B. (2008). Dysregulation of Adipose Glutathione Peroxidase 3 in Obesity Contributes to Local and Systemic Oxidative Stress. Mol. Endocrinol..

[58] Langhardt J., Flehmig G., Klöting N., Lehmann S., Ebert T., Kern M., Schön M.R., Gärtner D., Lohmann T., Dressler M. (2018). Effects of Weight Loss on Glutathione Peroxidase 3 Serum Concentrations and Adipose Tissue Expression in Human Obesity. Obes. Facts.

[59] Bosch-Sierra N., Grau-del Valle C., Hermenejildo J., Hermo-Argibay A., Salazar J.D., Garrido M., Navajas-Porras B., Sáez G., Morillas C., Bañuls C. (2024). The Impact of Weight Loss on Inflammation, Oxidative Stress, and Mitochondrial Function in Subjects with Obesity. Antioxidants.

[60] Bellanti F., Losavio F., Quiete S., Lo Buglio A., Calvanese C., Dobrakowski M., Kasperczyk A., Kasperczyk S., Vendemiale G., Cincione R.I. (2024). A Multiphase Very-Low Calorie Ketogenic Diet Improves Serum Redox Balance by Reducing Oxidative Status in Obese Patients. Free Radic. Biol. Med..

