# Supplementary material for: Very-Low-Energy Ketogenic Therapy Modulates the Metabolic–Antioxidant Axis in Patients with Obesity and Type 2 Diabetes: A Non-Randomized Clinical Trial
Source: Antioxidants (Basel). 2026 Jul 4;15(7):844. doi: 10.3390/antiox15070844 (PMC13404200; doi:10.3390/antiox15070844)
Supplement: Supplementary file 1 [file antioxidants-15-00844-s001.zip › antioxidants-4387301-supplementary.pdf]

Supplementary tables

Table S1

Medication	Group	Before enrollment	T0	T30	T90
Metformin	Control	8/10 (80%)	8/10 (80%)	8/10 (80%)	8/10 (80%)
	VLEKT	8/10(80%)	8/10 (80%)	8/10 (80%)	8/10 (80%)
	MedD	8/10(80%)	8/10 (80%)	8/10 (80%)	8/10 (80%)
GLP-1RA	Control	7/10 (70%)	7/10 (70%)	7/10(70%)	7/10 (70%)
	VLEKT	9/10 (90%)	9/10 (90%)	8/10 (80%)	8/10 (80%)
	MedD	7/10 (70%)	7/10 (70%)	7/10 (70%)	7/10 (70%)
SGLT2i	Control	3/10 (30%)	3/10 (30%)	3/10 (30%)	3/10 (30%)
	VLEKT	1/10 (10%)	0/10 (0.0%)	0/10 (0.0%)	0/10 (0.0%)
	MedD	2/10 (20%)	2/10 (20%)	2/10 (20%)	2/10 (20%)
Statins	Control	10/10 (100%)	10/10 (100%)	10/10 (100%)	10/10 (100%)
	VLEKT	10/10 (100%)	10/10 (100%)	10/10 (100%)	10/10 (100%)
	MedD	10/10 (100%)	10/10 (100%)	10/10 (100%)	10/10 (100%)

Table S1. Medication use before enrolment, at baseline and during follow-up according to intervention group. Data are reported as n/N (%). “Before enrolment” refers to medication use before any protocol-driven treatment modification. In the VLEKT group, SGLT-2 inhibitors were discontinued in one participant 7 days before baseline assessment (T0), according to safety procedures for ketogenic interventions. During follow-up, GLP-1RA therapy was discontinued in one participant in the VLEKT group because of gastrointestinal intolerance. Abbreviations: VLEKT, very low energy ketogenic therapy; MedD, Mediterranean diet; GLP-1RA, glucagon-like peptide-1 receptor agonists; SGLT2i, sodium-glucose cotransporter-2 inhibitors.

Table S2

Variable	*VLEKT vs Control [95% CI]*	*VLEKT vs MedD [95% CI]*
Weight (kg)	-15.17 [-18.25; -12.09]	-14.77 [-17.85; -11.69]
BMI (kg/m²)	-5.60 [-6.73; -4.47]	-5.42 [-6.55; -4.29]
WHtR	-0.083 [-0.113; -0.052]	-0.071 [-0.102; -0.041]
FM (kg)	-9.98 [-13.28; -6.68]	-10.92 [-14.26; -7.57]
FFM (kg)	-4.76 [-6.54; -2.98]	-3.68 [-5.49; -1.87]
PhA (°)	-0.06 [-0.52; 0.40]	0.03 [-0.43; 0.50]
ln(FPG)	-0.170 [-0.318; -0.023]	-0.250 [-0.398; -0.103]
HbA1c (%)	-0.68 [-1.29; -0.07]	-0.96 [-1.57; -0.35]
HOMA-IR	-6.90 [-9.87; -3.93]	-6.47 [-9.44; -3.50]
TC (mg/dL)	-5.20 [-25.51; 15.11]	-14.50 [-34.81; 5.81]
HDL (mg/dL)	-5.10 [-9.91; -0.29]	-5.10 [-9.91; -0.29]
LDL (mg/dL)	1.70 [-16.77; 20.17]	-2.32 [-20.79; 16.15]
ln(TG)	-0.052 [-0.320; 0.215]	-0.239 [-0.507; 0.028]

Table S2. Adjusted pairwise contrasts of changes from T0 to T90 for key outcomes across groups. Values represent adjusted between-group differences in estimated changes from baseline to study end, with 95% confidence intervals. Pairwise contrasts were derived from the same adjusted linear mixed-effects models used for the main analyses. Log-transformed variables are reported on the log scale. **Abbreviations:** VLEKT, very low-energy ketogenic therapy; MedD, Mediterranean diet; CI, confidence interval; BMI, body mass index; WHtR, waist-to-height ratio; FM, fat mass; FFM, fat-free mass; PhA, phase angle; FPG, fasting plasma glucose; HbA1c, glycated hemoglobin; HOMA-IR, homeostatic model assessment of insulin resistance; TC, total cholesterol; HDL, high-density lipoprotein cholesterol; LDL, low-density lipoprotein cholesterol; TG, triglycerides; LMM, linear mixed-effects model.

Table S3

Variable	Control β (95% CI)	p-value	VLEKT β (95% CI)	p-value	MedD β (95% CI)	p-value
Weight (kg)	-1.05 (-3.41, 1.31)	0.384	-16.22 (-18.58, -13.86)	**<0.001**	-1.45 (-3.81, 0.91)	0.229
BMI (kg/m²)	-0.30 (-1.16, 0.56)	0.494	-5.90 (-6.76, -5.04)	**<0.001**	-0.48 (-1.34, 0.38)	0.274
WHtR	0.00 (-0.02, 0.03)	0.781	-0.08 (-0.10, -0.06)	**<0.001**	-0.01 (-0.03, 0.02)	0.505
FM (kg)	-1.59 (-4.12, 0.94)	0.218	-11.57 (-14.10, -9.04)	**<0.001**	-0.65 (-3.26, 1.96)	0.624
Fat-free mass (kg)	0.54 (-0.83, 1.91)	0.440	-4.22 (-5.59, -2.85)	**<0.001**	-0.54 (-1.95, 0.87)	0.451
Phase angle (°)	-0.16 (-0.66, 0.34)	0.579	-0.16 (-0.38, 0.06)	0.374	-0.20 (-0.42, 0.02)	0.294
ln(FPG)	-0.05 (-0.16, 0.06)	0.394	-0.22 (-0.33, -0.11)	**<0.001**	0.03 (-0.08, 0.14)	0.594
HbA1c	-0.30 (-0.77, 0.17)	0.211	-0.98 (-1.45, -0.51)	**<0.001**	-0.02 (-0.49, 0.45)	0.933
HOMA-IR	0.78 (-1.50, 3.06)	0.502	-6.12 (-8.40, -3.84)	**<0.001**	0.35 (-1.93, 2.63)	0.764
TC (mg/dL)	-13.50 (-29.08, 2.08)	0.089	-18.70 (-34.28, -3.12)	**0.019**	-4.20 (-19.78, 11.38)	0.597
ln(TG/HDL)	-0.20 (-0.43, 0.03)	0.091	-0.15 (-0.38, 0.08)	0.194	-0.01 (-0.24, 0.22)	0.927
HDL (mg/dL)	0.50 (-3.10, 4.10)	0.786	-4.60 (-8.20, -1.00)	**0.012**	0.50 (-3.10, 4.10)	0.786
LDL (mg/dL)	-8.94 (-23.11, 5.23)	0.216	-7.24 (-21.41, 6.93)	0.317	-4.92 (-19.09, 9.25)	0.496
ln(TG) (mg/dL)	-0.20 (-0.41, 0.00)	0.056	-0.25 (-0.46, -0.05)	**0.016**	-0.01 (-0.22, 0.19)	0.897
SOD (inh. rate)	4.46 (0.72, 8.21)	0.020	6.18 (2.44, 9.93)	**0.001**	-0.72 (-4.47, 3.03)	0.705
GPx (nmol)	66.78 (32.04, 101.52)	<0.001	-21.16 (-55.90, 13.58)	0.233	6.22 (-28.52, 40.96)	0.726
ln(NLR)	-0.11 (-0.32, 0.11)	0.325	-0.10 (-0.33, 0.12)	0.364	0.13 (-0.11, 0.37)	0.277
ln(IL-6)	-0.28 (-0.57, 0.01)	0.060	-0.24 (-0.53, 0.05)	0.098	-0.08 (-0.37, 0.21)	0.587
ln(CXCL5/ENA-78)	-0.084 (-0.190, 0.022)	0.119	-0.057 (-0.163, 0.049)	0.290	-0.068 (-0.174, 0.038)	0.210
IL-18	32.0 (-65.7, 129.8)	0.521	-103.2 (-212.5, 6.0)	0.064	-38.8 (-142.8, 65.2)	0.465

Table S3. Model-adjusted within-group changes from baseline in anthropometric, metabolic, antioxidant, and inflammatory parameters. Data are presented as regression coefficients (β) with 95% confidence intervals derived from linear mixed-effects models adjusted for baseline value, age and sex. The β coefficients represent the estimated change from baseline to study end within each group. P values refer to within-group changes over time. Statistical significance was defined as $p < 0.05$, and significant values are highlighted in bold. Variables with skewed distributions were natural log-transformed and are presented as β coefficients on the log scale. For log-transformed variables, β coefficients can be interpreted as relative (percentage) changes. **Abbreviations:** VLEKT, very low energy ketogenic therapy; MedD, Mediterranean diet; FM, fat mass; FFM, fat-free mass; WHtR, waist-to-height ratio; FPG, fasting plasma glucose; TC, total cholesterol; TG/HDL, triglyceride-to-HDL ratio; HOMA-IR, homeostasis model assessment of insulin resistance; SOD, superoxide dismutase; GPx, glutathione peroxidase; NLR, neutrophil-to-lymphocyte ratio; IL-6, interleukin-6; TNF-α, tumour necrosis factor alpha; MCP-1/CCL2, monocyte chemoattractant protein-1; CXCL5/ENA-78, C-X-C motif chemokine ligand 5.

Table S4

**Group**	**PREDIMED T0**	**PREDIMED T90**	**Δ PREDIMED**	**p-value**
MedD	6.5 ± 2.0	8.6 ± 2.1	+2.1 ± 2.2	**0.020**
Control	6.6 ± 2.1	8.7 ± 1.6	+2.1 ± 2.2	**0.023**

Table S4. Changes in PREDIMED score in the MedD and control groups. Values are reported as mean ± SD. Δ PREDIMED represents the within-group change from baseline to T90. P values refer to within-group changes over time. Statistical significance was defined as $p < 0.05$, and significant values are highlighted in bold . ***Abbreviations***: MedD, Mediterranean diet;

Table S5

MedD subject	Prescribed diet target	Deviation from target	Energy adherence ±10%
Subject 1	1700 kcal/day	-12.0%	No
Subject 2	1900 kcal/day	-19.8%	No
Subject 3	1800 kcal/day	-17.0%	No
Subject 4	1700 kcal/day	-3.7%	Yes
Subject 5	1700 kcal/day	-10.4%	No
Subject 6	1200 kcal/day	+30.7%	No
Subject 7	900 kcal/day	+4.4%	Yes
Subject 8	1300 kcal/day	+19.7%	No
Subject 9	1100 kcal/day	+66.6%	No
Subject 10	1200 kcal/day	+18.7%	No

Table S5. Energy adherence to the prescribed Mediterranean dietary intervention at T90. The individualized prescribed dietary target was estimated as basal metabolic rate assessed by Tanita MC-780 MA minus 500 kcal/day and rounded to the nearest 100 kcal. Adherence was defined as a reported energy intake within ±10% of the prescribed target based on 24-hour dietary recall data.

Table S6

Control group	Baseline energy intake	Deviation from baseline	Energy deviation ±10%
Subject 1	1071 kcal/day	+17.5%	No
Subject 2	969 kcal/day	+3.5%	Yes
Subject 3	1492 kcal/day	-17.9%	No

Subject 4	1194 kcal/day	+21.6%	No
Subject 5	2401 kcal/day	-8.0%	Yes
Subject 6	1952 kcal/day	-2.7%	Yes
Subject 7	1432 kcal/day	+5.0%	Yes
Subject 8	1418 kcal/day	+25.7%	No
Subject 9	1240 kcal/day	-16.5%	No
Subject 10	1718 kcal/day	+40.6%	No

Table S6. Energy intake variation relative to baseline in the control group at T90. The estimated daily energy intake at baseline was used as the individual reference value for each participant. Deviation was calculated as the percentage difference between energy intake at T90 and baseline energy intake based on 24-hour dietary recall data. A ±10% threshold was used to identify participants with substantially unchanged reported energy intake over the study period.

Table S7

Antioxidant marker	Metabolic parameter	ρ	p-value	n
ΔSOD	ΔFM (kg)	-0.29	0.124	29
ΔSOD	ΔHOMA-IR	-0.36	0.058	29
ΔSOD	ΔWeight (kg)	-0.24	0.201	29
ΔSOD	ΔTriglycerides (mg/dL)	-0.23	0.229	29
ΔGPx	ΔFM (kg)	0.27	0.159	29
ΔGPx	ΔHOMA-IR	0.42	**0.024**	29
ΔGPx	ΔWeight (kg)	0.37	**0.049**	29
ΔGPx	ΔTriglycerides (mg/dL)	-0.14	0.466	29

Table S7. Spearman correlations of ΔSOD and ΔGPx with metabolic parameters. Spearman correlation coefficients (ρ), p-values and number of observations are reported for the associations between changes in antioxidant markers and changes in metabolic and **body** composition parameters. Analyses were performed using absolute changes from T0 to T90. Statistical significance was defined as $p < 0.05$, and significant values are highlighted in bold. **Abbreviations:** Δ, change from T0 to T90; SOD, superoxide dismutase; GPx, glutathione peroxidase; FM, fat mass; HOMA-IR, homeostasis model assessment of insulin resistance.

Table S8

Variable	β (95% CI)	p-value
Δfat mass (kg)	-0.31 (-0.94 to 0.32)	0.319
ΔHOMA-IR	-0.47 (-0.91 to -0.03)	0.036
VLEKT vs Control/MedD	-9.33 (-19.15 to 0.48)	0.061

Table S8. Multivariable linear regression analysis assessing factors associated with changes in SOD. Intervention group was modelled as VLEKT versus Control/MedD combined. The model included changes in fat mass, changes in HOMA-IR, intervention group, age, sex and baseline SOD. Data are presented as regression coefficients (β) with 95% confidence intervals. Robust standard errors were applied. **Abbreviations:** SOD, superoxide dismutase; HOMA-IR, homeostasis model assessment of insulin resistance; VLEKT, very low energy ketogenic therapy; MedD, Mediterranean diet; CI, confidence interval.

Table S9

Variable	β (95% CI)	p-value
Δfat mass (kg)	-1.46 (-6.11 to 3.20)	0.522
ΔHOMA-IR	1.06 (-4.11 to 6.22)	0.676
VLEKT vs Control/MedD	-17.74 (-120.37 to 84.89)	0.723

Table S9. Multivariable linear regression analysis assessing factors associated with changes in GPx. Intervention group was modelled as VLEKT versus Control/MedD combined. The model included changes in fat mass, changes in HOMA-IR, intervention group, age, sex and baseline GPx. Data are presented as regression coefficients (β) with 95% confidence intervals. Robust standard errors were applied. **Abbreviations**: GPx, glutathione peroxidase; HOMA-IR, homeostasis model assessment of insulin resistance; VLEKT, very low energy ketogenic therapy; MedD, Mediterranean diet; CI, confidence interval.

Table S10

Variable	n	Spearman's ρ	p-value
Δ kcal vs Δ SOD	30	-0.060	0.750
Δ kcal vs Δ GPx	30	0.444	**0.015**

Table S10. Association between changes in energy intake and antioxidant enzyme activity. Associations were assessed using Spearman's rank correlation. Changes in energy intake were calculated as the deviation from the group-specific dietary reference: the 800 kcal/day target for VLEKT, the individualized prescribed caloric target for MedD, and baseline energy intake for the control group. Statistical significance was defined as $p < 0.05$, and significant values are highlighted in bold. **Abbreviations:** Δ, change from T0 to T90; SOD, superoxide dismutase; GPx, glutathione peroxidase.
